# Diameter Dependent Melting and Softening of dsDNA Under Cylindrical Confinement

**DOI:** 10.3389/fchem.2022.879746

**Published:** 2022-05-02

**Authors:** Khadka B. Chhetri, Chandan Dasgupta, Prabal K. Maiti

**Affiliations:** ^1^ Center for Condensed Matter Theory, Department of Physics, Indian Institute of Science, Bangalore, India; ^2^ Department of Physics, Prithvinarayan Campus, Tribhuvan University, Pokhara, Nepal

**Keywords:** chirality indices, denaturation, bonded and non-bonded interactions, potential map, persistence length, hydrophobic surface, torsional stiffness, stretch modulus

## Abstract

Carbon nanotubes (CNTs) are considered promising candidates for biomolecular confinement, including DNA encapsulation for gene delivery. Threshold values of diameters have been reported for double-stranded DNA (dsDNA) encapsulation inside CNTs. We have performed all-atom molecular dynamics (MD) simulations of dsDNAs confined inside single-walled CNTs (SWCNTs) at the physiologically relevant temperature of 300 K. We found that the dsDNA can be confined without being denatured only when the diameter of the SWCNT exceeds a threshold value. Below this threshold diameter, the dsDNA gets denatured and melts even at the temperature of 300 K. Our simulations using SWCNTs with chirality indices (20,20) to (30,30) at 300 K found the critical diameter to be 3.25 nm (corresponding to (24,24) chirality). Analyses of the hydrogen bonds (H-bonds), Van der Walls (VdW) energy, and other inter-base interactions show drastic reduction in the number of H-bonds, VdW energy, and electrostatic energies between the bases of dsDNA when it is confined in narrower SWCNTs (up to diameter of 3.12 nm). On the other hand, the higher interaction energy between the dsDNA and the SWCNT surface in narrower SWCNTs assists in the melting of the dsDNA. Electrostatic mapping and hydration status analyses show that the dsDNA is not adequately hydrated and the counter ion distribution is not uniform below the critical diameter of the SWCNT. As properly hydrated counter ions provide stability to the dsDNA, we infer that the inappropriate hydration of counter ions and their non-uniform distribution around the dsDNA cause the melting of the dsDNA inside SWCNTs of diameter below the critical value of 3.25 nm. For confined dsDNAs that do not get denatured, we computed their elastic properties. The persistence length of dsDNA was found to increase by a factor of about two and the torsional stiffness by a factor of 1.5 for confinement inside SWCNTs of diameters up to 3.79 nm, the stretch modulus also following nearly the same trend. Interestingly, for higher diameters of SWCNT, 3.79 nm and above, the dsDNA becomes more flexible, demonstrating that the mechanical properties of the dsDNA under cylindrical confinement depend non-monotonically on the confinement diameter.

## 1 Introduction

In the modern field of biotechnology, single-walled carbon nanotubes (SWCNTs) with diameters in the nanometer range are proposed as gene delivery vehicles. The ease of diversified functionalization has allowed SWCNTs to be the most adaptable non-viral vector for gene therapy (Singh et al., 2020). Not only SWCNTs but also multi-walled nanotubes (MWCNTs) are found to be effective for gene delivery as well as drug delivery (Ji et al., 2010). Along with biocompatibility and high drug loading capacity, SWCNTs have the ability to release therapeutic agents at targeted sites (Lakshminarayanan et al., 2013; Lakshminarayanan et al., 2015; Zare et al., 2021). SWCNTs have a wider hydrophobic surface area that has flexible interaction with cargo (Cruz et al., 2014; Zare et al., 2021). Along with these attractive features, SWCNTs also have some negative aspects such as lack of biodegradability and toxicity. In spite of these minimal adverse effects, SWCNTs have been investigated for a diversity of applications, including the delivery of nucleic acids, due to their unique properties, such as easy chemical functionalization, propensity to act as a template, and many more (Matyshevska et al., 2001).

Gene delivery introduces foreign genetic material, such as DNA or RNA, into host cells. The cell membrane is a bilayer of phospholipids with net negative charge, along with proteins embedded in it. So, the cell membrane functions as an impermeable barrier for molecules like DNA and RNA that have negatively charged phosphate backbones. Several techniques have been developed to permeate the nucleic acids through the cell membrane, including packing nucleic acids inside a physical structure to create transient pores in the membrane to introduce the DNA directly into the host cells (Lasic et al., 1997; Vasumathi and Maiti, 2010; Nandy and Maiti, 2011; Zhang et al., 2012). SWCNTs are expected to be efficient candidates to be used as nanopores for biomolecular confinement. They have been proposed as future templates for DNA encapsulation due to their hydrophobic nature (Cruz et al., 2015).

Numerous experimental, theoretical, and simulation works have been performed to study the encapsulation of DNA inside SWCNTs and its physical properties under encapsulation. The experimental work by Geng et al. (Geng et al., 2014) has shown that short SWCNTs can be spontaneously inserted into lipid bilayers and they remain in cell membranes to form channels that can easily transport water, protons, small ions, and DNA into the cell. Simulation work of Gao et al. (2003) found that a DNA molecule can be spontaneously inserted into a SWCNT in a water solute environment. Mogurampelly et al. (Mogurampelly and Maiti, 2013) found the critical diameter of SWCNT for the translocation of a small interfering RNA (siRNA), a double-stranded RNA (dsRNA) with 21–23 nucleotides, to be 2.4 nm, whereas double-strand DNA (dsDNA) of the same sequence cannot translocate inside the SWCNT due to a sizeable free energy barrier. They established that for the encapsulation of dsDNA and siRNA onto SWCNTs, the critical diameters are ∼ 2.7 nm and ∼ 2.4 nm, respectively, below which confinement is completely inhibited due to a large free-energy barrier at the nanopore entrance. Nandy et al. (2012) found that single-stranded DNA (ssDNA) strongly adsorbs and also translocates into a carbon nanotube, whereas dsDNA only adsorbs but does not translocate into SWCNT of chirality indices (20,20). They found the unzipping of a few base pairs of dsDNA that are in contact with the SWCNT end, and the subsequent binding of the broken base pairs to the SWCNT surface prevents the dsDNA from entering the nanotube.

Molecular Dynamics (MD) simulations of Cruz et al. (Cruz et al., 2014; Cruz et al., 2015) observed the spontaneous encapsulation of dsDNA in the 4 nm nanopore of a SWCNT, but not when the diameter narrowed down to 3 nm. They reported that this behavior is due to the confined dsDNA termini directly contacting the hydrophobic walls of the SWCNT with no solvent slab in-between. The numerical modeling work of Alshehri (Alshehri, 2021) has shown that the radius required for encapsulating dsDNA is > 1.28 nm for different nanopores, including SWCNT. In another numerical study of Alshehri et al. (Alshehri and Hill, 2017), the suction behavior of the SWCNT was observed to depend on the radius of the SWCNT. They predicted that the dsDNA molecule can be inserted into a SWCNT for radii greater than 1.30 nm, and estimated the optimal radius of the SWCNT to be ∼1.36 nm for completely encapsulating the dsDNA, which corresponds to chirality indices (20, 20). They found the difference between the radii of the SWCNT and the dsDNA at the point of least interaction between their surfaces to be approximately 3.25 Å (Alshehri et al., 2012). The numerical modelings mentioned above do not include explicit solvent. In an aqueous environment, there should be a larger radii difference, since, for nanotubes dissolved in water, there are additional Van der Waals, hydrophobic, and hydrogen bonding interactions in the nanotube/water/DNA complex (Xue and Chen, 2005). Previous research has shown that the biomolecules can be confined in SWCNTs with 2.7 nm radius and MWCNTs with 3–4 nm radius in a temperature range of 350–400 K (Gao and Kong, 2004; Iijima et al., 2005; Ghosh et al., 2009). Such high temperatures are physiologically irrelevant. Cruz et al. (Cruz et al., 2014; Cruz et al., 2015) and Nandy et al. (2012) found that the contact between a dsDNA segment and the hydrophobic wall of a SWCNT results in some rearrangement of the H-bonds between the nucleobases and some of the base pairs that come on the contact of the SWCNT wall are melted. Jonchhe et al. (2020) found that a DNA hairpin, when enclosed inside a nanocage, severely loses its mechanical and thermodynamic stability. Different research works have explored the melting of dsDNA in confined geometry (Singh et al., 2009; Kumar and Li, 2010; Singh et al., 2010; Mohanta et al., 2019; Chauhan and Kumar, 2021). These results reveal that it is possible to encapsulate a dsDNA inside SWCNT/MWCNTs, but its stability is weakened, and these properties are dependent crucially on the nanotube diameter.

Our focus in this study is not on the encapsulation of dsDNA inside SWCNTs, since various researchers have already explored this process. We have simulated a dsDNA confined inside a SWCNT and studied its physical and mechanical properties in this confined geometry. For this, we have considered the physiologically relevant temperature of 300 K. We have carried out all-atom MD simulations of the dsDNA confined in SWCNTs of different diameters corresponding to the chirality indices (20,20) to (30,30). We analyzed various physical properties of the dsDNA such as Watson-Crick hydrogen-bonds (WC H-bonds), non-bonded electrostatic and Van der Waals (VdW) energy profile and hydration status. When the confined dsDNA is not denatured, we have computed mechanical properties such as stretch modulus, persistence length, and torsional stiffness. The main purpose of our study is to examine how these properties of the dsDNA are affected by the strong confinement provided by the SWCNT, and how the effects of confinement depend on the diameter of the SWCNT.

This article is organized as follows. We begin with the “Materials and Methods” section that contains the details of the dsDNA and SWCNT model building and the all-atom MD simulations. Details of the bonded and non-bonded energy and mechanical properties computation are also discussed in this section. In the results section, we describe the outputs of our computation of different properties of the confined dsDNA and discuss the physical changes of the dsDNA caused by confinement. Finally, the results are summarized along with some future perspectives.

## 2 Materials and Methods

### 2.1 Simulation Details

We performed all-atom MD simulations using Amber18 software package (Case et al., 2018). The dsDNA duplex (d[GTCGCGAATTCGCGAC]) was prepared using the nucleic acid builder (NAB) (Macke and Case, 1997) tool. The SWCNTs were modeled using the nanotube builder module of the visual molecular dynamics (VMD) (Humphrey et al., 1996; Hsin et al., 2008) software. The dsDNA was placed inside SWCNTs using the xleap tool of the Amber18 package (Case et al., 2018). These individual complex structures were solvated using the TIP3P water model (Jorgensen et al., 1983; Mark and Nilsson, 2001) in such a way to ensure 12 Å solvation shell in all the three directions. As the phosphate backbone of dsDNA contains negative charges, the systems were made charge-neutral by adding Na+ counter ions. To describe the interactions involving dsDNA, the DNA.OL15 (Zgarbová et al., 2015; Galindo-Murillo et al., 2016) force field was used. General AMBER Force Field (GAFF2) (Vassetti et al., 2019) was used to describe the interaction parameters for the SWCNT. To describe the interactions involving Na+ counter ions, the Joung–Cheatham ion parameter set (Joung and Cheatham, 2008) was used.

The solvated systems were then energy minimized using the steepest descent algorithm (2,500 steps) followed by the conjugate gradient algorithm (2,500 steps). The systems were then equilibrated, allowing the ions and water molecules to redistribute from their original ordered condition. All of the solute atoms (nucleic acid + SWCNT atoms) were held fixed using a harmonic potential with force constant of 500 kcal mol^−1^Å^−2^ to eliminate any bad contacts. After that, the positional restrain on solute atoms was decreased to zero in five steps, each having 5,000 energy minimization steps. The systems were then heated from 10 to 300 K in 4 stages. The heating stages involved: 10–50 K for 6,000 steps, 50–100 K for 12,000 steps, 100–200 K for 10,000 steps, and 200–300 K for 12,000 steps. Harmonic constraints with force constant of 20 kcal mol^−1^Å^−2^ were used to keep the solute particles in their positions. The systems were then finally equilibrated for 10,000 steps without any restraint. During heating, the temperature was controlled using the Langevin thermostat (Van Gunsteren and Berendsen, 1988; Davidchack et al., 2009) with a coupling constant of 0.5 ps. After heating, the systems were simulated for 2 ns using an NPT ensemble. Berendsen weak coupling method (Berendsen et al., 1984; Hünenberger, 2005) with a coupling constant of 0.5 ps was used to maintain a pressure of 1 atm. Finally, 200 ns long simulations were conducted in the NVT ensemble. During the 200 ns long NVT production run, the SWCNT was position restrained using a force constant of 20 kcal mol^−1^Å^−2^. During simulation, all bonds involving hydrogens were constrained using the SHAKE algorithm (Ryckaert et al., 1977). All simulations utilized a 2 fs time integration step. The Particle Mesh Ewald (PME) (Darden et al., 1993) technique was used to model long-range Coulomb interactions. For this, a real-space cut-off of 10 Å was chosen. At the cut-off, the VdW and real space part of the electrostatic interactions were truncated. Several of our earlier simulation works employing DNA and DNA-based nanostructures have effectively adopted similar modelling approaches (Maiti et al., 2004; Maiti et al., 2006; Garai et al., 2015; Garai et al., 2018; Naskar et al., 2020; Naskar and Maiti, 2021; Chhetri et al., 2022a).

As described above, for various chirality indices of SWCNT [(20,20) to (30,30), corresponding to diameters 2.71–4.07 nm], we ran eight separate simulations. In addition to it, we also did a simulation of a dsDNA without SWCNT. So, in total, nine different simulations for dsDNA were carried out. Using VMD (Humphrey et al., 1996) software, the simulated structures were viewed, and the images displayed were created. We employed the 3DNA (Lu and Olson, 2003) program to compute the helical properties of the dsDNA. The bending angle distribution, contour length distribution, and twist angle distribution were computed from the 3DNA output. These distributions were used to compute the dsDNA’s persistence lengths, stretch modulus, and torsional stiffness. In the analyses for H-bonds, bonded and non-bonded interactions, and helical parameters, we removed one base pair from each end of the simulated molecules to avoid the end-terminal effect.

### 2.2 Bonded and Non-Bonded Analysis

The hydrogen bonds (H-bonds) and the non-bonded (NB) interactions between the bases help to maintain the two anti-parallel DNA strands in duplex form. So, their analysis is critical to predict the stability of the dsDNA duplex. In this work, we used VMD (Humphrey et al., 1996) software to analyze the H-bonds between the base pairs of dsDNA, taking a distance cut-off of 3.5 Å and angle cut-off of 120° as suggested by IUPAC (Arunan et al., 2011; Naskar et al., 2019).

Along with the H-bonds of the dsDNA duplex, we computed non-native contacts between the dsDNA surface and the wall of the SWCNT using the CPPTRAJ (Roe and Cheatham, 2013) program, implemented with the Amber software package. For this computation, the initially minimized structure was taken as the reference time frame, and the distance cut-off between the dsDNA surface and the SWCNT wall was taken to be 3.0 Å. The number of non-native contacts at a given time frame is the difference between the number of contacts at the given time frame and the number of contacts at the reference time frame. The number of H-bonds and the number of non-native contacts help to determine the degree of melting of the base pairs in the dsDNA helix.

NB interaction energies (VdW and electrostatic) and bonded dihedral interaction energy of the bases of dsDNA were computed using the NAMD Energy (MDEnergy) module (Kalé et al., 1999) interfaced with VMD software. Similarly, the pair-wise interactions between the whole dsDNA and the SWCNT, as well as the end bases and the SWCNT, were computed to determine the physical or surface area contact between the dsDNA and the SWCNT.

Along with the different bonded and non-bonded interaction energies, we studied the hydration status of the confined dsDNA. If the dsDNA becomes dehydrated due to different physical environment, it can be denatured and melted (Svaren et al., 1987; Ghoshdastidar and Senapati, 2018). In other words, counter ions that are more hydrated stabilize dsDNA better than those that are less hydrated (Piškur and Rupprecht, 1995; Maiti and Bagchi, 2006). Narrower SWCNTs show apparently stronger hydrophobic nature (Kyakuno et al., 2016). So, the smaller diameter and strong hydrophobic nature of narrower SWCNTs can cause dehydration of the dsDNA. To know the hydration status of the dsDNA inside the SWCNT, we computed the number of water molecules inside a cylindrical water shell surrounding the dsDNA as well as the axial distribution of water molecules inside the SWCNT. The presence of counter-ions around a dsDNA strand also enhances its stability as counter-ions help in keeping the helix stable (Tan and Chen, 2006). With this in consideration, average electrostatic maps around the SWCNT were obtained to examine if counter-ions surrounding the dsDNA is distributed isotropically or not.

For dsDNAs confined inside SWCNT without significant denaturation, we computed their mechanical properties to examine how the cylindrical confinement affects these properties. We computed the persistence length, stretch modulus, and torsional stiffness to test the confined molecule’s bending, stretching, and twisting rigidity.

### 2.3 Mechanical Properties

#### 2.3.1 Persistence Length

The bending persistence length of a dsDNA is a basic mechanical property that quantifies its bending stiffness, taking dsDNA as a polymer analog. In this work, the bending persistence length (*l*_*p*_) was calculated from the bending angle distribution.

**FIGURE 1:**
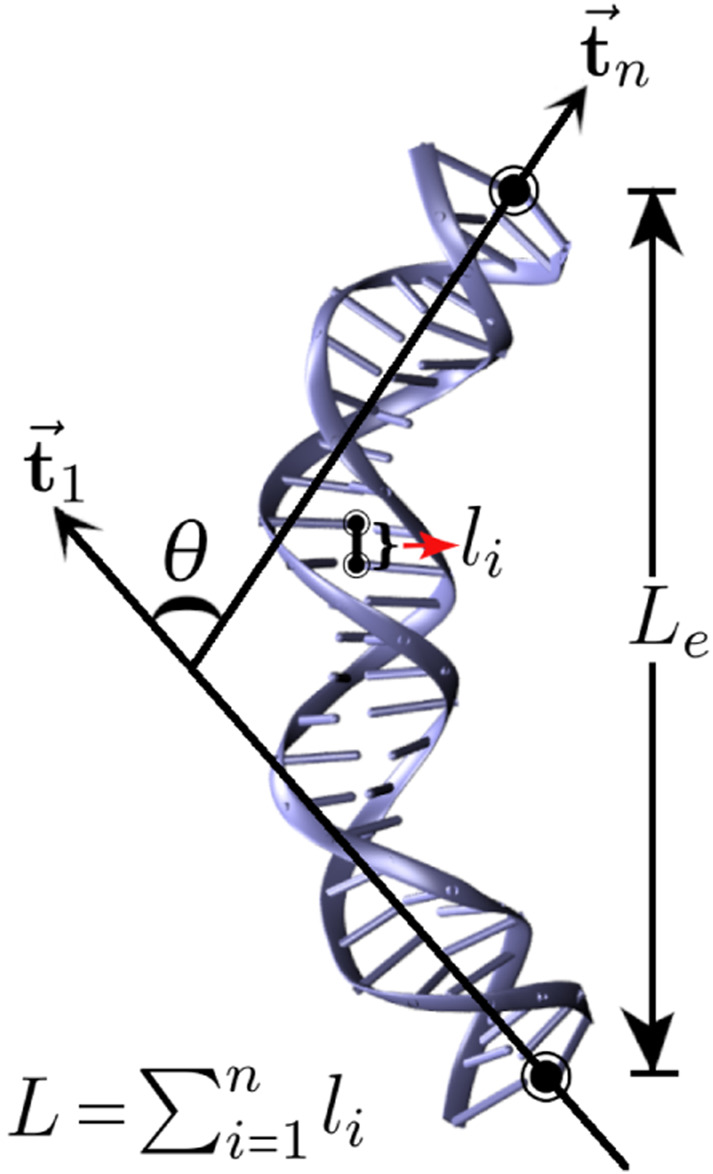
Schematic representation of a dsDNA where L_*e*_ represents its end-to-end distance, L represents its contour length, and *θ* represents the bending angle, the angle between unit vectors ${\vec{t}}_{1}$ and ${\vec{t}}_{n}$ .

To calculate the bending angle of the molecule, we look at the *ith* base pair of the molecule and the local unit vector ${\vec{t}}_{i}$ along the contour length (see Figure 1). Then, the bending angle of a molecule (*n* base pairs long) is given by $\theta =\cos^{-1}\left({\vec{t}}_{1}.{\vec{t}}_{n}\right)$ . In Figure 1, the quantity *L* is the contour length and *L*_*e*_ is the end-to-end distance of the molecule. If we consider the distance between the centers of mass of consecutive base pairs to be *l*_*i*_, then the average contour length is $L_{0}=\left\langle \sum_{i=1}^{n}l_{i}\right\rangle$ , where, ⟨⟩ represents a time average over all frames.

The bending angle distribution of a nucleic acid can be approximated by a Gaussian distribution, given by (Mogurampelly et al., 2013)

$$P\left(\theta \right)=\sqrt{\frac{\beta \kappa }{2\pi L_{0}}}e^{-\frac{\beta \kappa }{2L_{0}}\theta^{2}}$$ (1)

For small *θ*, we can rewrite Eq. 1 as:

$$\ln P\left(\theta \right)=-\frac{l_{p}}{L_{0}}\left(1-\cos\left(\theta \right)\right)+\frac{1}{2}\ln\left(\frac{\beta \kappa }{2\pi L_{0}}\right)$$ (2)

where, the bending modulus(*κ*) is given by: $\kappa =\frac{l_{p}}{\beta }$ = *k*_*B*_ *Tl*_*p*_, *k*_*B*_ being the Boltzmann’s constant (Garai et al., 2018; Naskar et al., 2019; Aggarwal et al., 2020). The bending persistence length is extracted from the slope of the graph of ln *P*(*θ*) versus (1 − cos *θ*) (Mazur, 2007).

#### 2.3.2 Stretch Modulus

The stretch modulus of dsDNA is a basic mechanical property that quantifies its resistance to being deformed elastically under tensile stress. In this work, the stretch modulus (*γ*_*G*_) was calculated from the contour length distribution. Similar to the bending angle distribution, one can describe the length fluctuation by a Gaussian probability distribution, given by (Garai et al., 2015).

$$P\left(L\right)=\sqrt{\frac{\beta \gamma_{G}}{2\pi L_{0}}}e^{-\frac{\beta \gamma_{G}}{2L_{0}}{\left(L-L_{0}\right)}^{2}}$$ (3)

By taking logarithm, Eq. 3 can be rewritten as:

$$\ln P\left(L\right)=-\frac{\beta \gamma_{G}L_{0}}{2}{\left(\frac{L}{L_{0}}-1\right)}^{2}+\frac{1}{2}\ln\frac{\beta \gamma_{G}}{2\pi L_{0}}$$ (4)

where *L*_0_ is the time-averaged contour length, *γ*_*G*_ is the stretch modulus, and *β* = 1/*k*_*B*_ *T* with *T* being the temperature of the equilibrated system and *k*_*B*_ being the Boltzmann constant. The stretch modulus is extracted from the slope of the graph of ln *P*(*L*) versus ${\left(\frac{L}{L_{0}}-1\right)}^{2}$ .

#### 2.3.3 Torsional Stiffness

The twisting rigidity or torsional stiffness of a DNA molecule is a basic mechanical property that quantifies its resistance to torsional deformation. The torsional modulus or torsional stiffness (*C*) of dsDNA can be obtained from the inverse of the diagonal element of the covariance matrix and is given by (Bryant et al., 2003; Nomidis et al., 2017)

$$C=\frac{k_{B}TL}{\sigma_{\phi }^{2}}$$ (5)

where *L* and *ϕ* are the two global helical coordinates, the sum of the helical rise (H-rise) and the sum of the helical twist (H-twist) of each base pair, respectively, and $\sigma_{\phi }^{2}$ is the variance of the angle *ϕ*. The torsional persistence length is given by $\frac{L}{{\sigma_{\phi }}^{2}}$ (Kriegel et al., 2017; Skoruppa et al., 2017).

## 3 Results and Discussion

The dsDNA has a diameter of about 2 nm. Therefore, we have taken different SWCNTs with chirality indices starting from (20,20), which is slightly wider to accommodate the dsDNA inside it. The chirality indices (*p*, *q*) of an SWCNT and its diameter *d* in *nm* are related as:

$$d=0.0783\sqrt{{\left(p+q\right)}^{2}-\left.p\times q\right)}$$ (6)

Table 1 presents the diameters of SWCNTs corresponding to different chirality indices.

**TABLE 1:** Diameters corresponding to different chirality indices of SWCNTs.


Chirality Indices (*p*, *q*)	(20,20)	(22,22)	(23,23)	(24,24)	(25,25)	(26,26)	(28,28)	(30,30)
Diameter in *nm*	2.71	2.98	3.12	3.25	3.39	3.52	3.79	4.07

### 3.1 Structure of the Confined dsDNA

**FIGURE 2:**
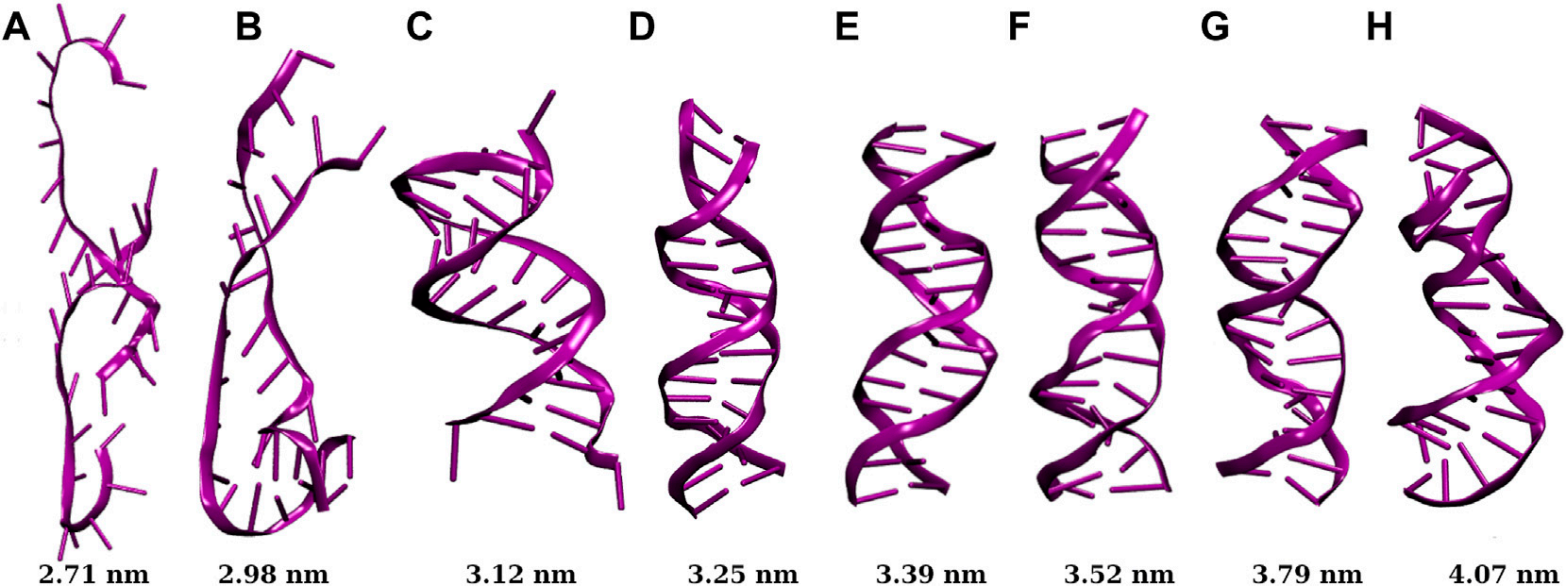
Snapshots of dsDNA confined inside SWCNTs of different chirality indices, where **(A,B,C,D,E,F,G,H)** correspond to chirality indices (20,20), (22,22), (23,23), (24,24), (25,25), (26,26), (28,28), and (30,30) respectively. The value below each snapshot is the diameter of the corresponding SWCNT. Below chirality indices (24,24) or 3.25 nm diameter of SWCNT, the dsDNA gets denatured with a significant cut-off of H-bonds.

Instantaneous snapshots of dsDNA confined inside SWCNTs of different chirality indices are shown in Figure 2. We see that the dsDNA confined inside the SWCNT is highly denatured when the diameter of the SWCNT is 3.12 nm or lower (corresponding to chirality indices (23,23) or less). For diameter 3.25 nm or above (corresponding to chirality indices (24,24) or above), the dsDNA is confined inside the nanopore of SWCNT without being denatured.

The root mean square deviation (RMSD) of the backbone atoms of dsDNA duplexes with respect to their initially minimized structures are plotted in Figure 4A. The RMSD of a structure at time t with regard to its reference structure is given by: $RMSD\left(t\right)=\sqrt{\frac{1}{N}\sum_{i=1}^{N}\|x_{i}\left(t\right)-x_{i}^{ref}}{\|}^{2}$ , where {*x*_*i*_(*t*)} are the coordinates of the N atoms of the structure and $\left\{x_{i}^{ref}\right\}$ are the corresponding coordinates of the energy minimized structure. The RMSD plots show larger deviations of the backbone atoms when the dsDNA is confined inside a SWCNT of chirality indices (23,23) or less. For chirality indices (24,24) or above, the RMSD is found to be about 1.8 Å on the average, which roughly matches with that of unconfined dsDNA, i.e., the bulk dsDNA. Below (23,23) chirality indices, the RMSD increased sharply.

The root mean square fluctuation (RMSF) gives a measure of individual residue’s flexibility during the simulation run. The RMSF of *ith* residue of a structure is given by: $RMSF\left(i\right)=\sqrt{\frac{1}{T}\sum_{t_{j}=1}^{T}\|x_{i}\left(t_{j}\right)-\bar{x_{i}}{\|}^{2}}$ , where *x*_*i*_ (*t*_*j*_) is the position of the residue at time *t*_*j*_, $\bar{x_{i}}$ is its time-averaged position, and *T* is the total time over which averaging is done. The RMSF plots in Figure 4B also show that the dsDNA inside narrower SWCNTs with diameter less than 3.25 nm (corresponding to chirality indices (24,24)) has a higher degree of disorder of most of the residue atoms. However, the dsDNA inside SWCNTs of diameter 3.25 nm or above shows highly restricted flexibility except those of the end bases.

These features of RMSD and RMSF plots have made easier for us to understand the trends of denaturation of the dsDNA under cylindrical confinement. The dsDNA gets severely denatured if confined inside SWCNTs of chirality indices below (24,24). This implies that the threshold value of the diameter of the SWCNT for proper confinement of dsDNA without being denatured corresponds to the diameter for chirality indices (24,24), i.e. 3.25 nm.

### 3.2 Bonded and Non-bonded Analysis

For a detailed study of the physical situation of a dsDNA confined inside SWCNTs of different diameters, we have analyzed a few bonded and non-bonded physical quantities like the number of H-bonds, VdW energy, electrostatic energy, and dihedral energy that are responsible for providing structural stability of dsDNA.

The stability of the DNA double helix is determined by a delicate balance of interactions, including hydrogen bonds, hydration status of the duplex, and base-stacking interactions between the bases. Since base stacking is significantly more common in duplexes than in a single strand, inter-strand hydrogen bonding and strong stacking interactions between bases are important factors to duplex stability. Both inter-strand and intra-strand base-stacking interactions occur in nucleic acid duplexes. H-bonds and base stacking provide different levels of stability depending on the sequences. Nearest neighbor base-stacking interactions are major drivers of duplex stability because certain combinations of base pairs create more stable interactions than others.

**FIGURE 3:**
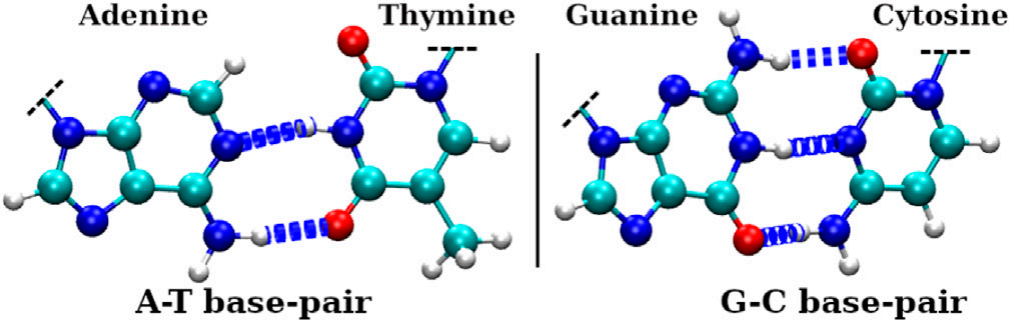
H-bonds involved in Watson-Crick (WC) base pairs of dsDNA, where left panel shows H-bonds between adenine(A) and thymine(T) bases and the right panel shows H-bonds between guanine(G) and cytosine(C) bases. The larger dark silver spheres, smaller bright silver spheres, blue spheres, and red spheres represent carbon, hydrogen, nitrogen, and oxygen atoms, respectively.

Our inter-strand hydrogen bond analysis shown in Figure 5A showed a drastic reduction in its average number when the dsDNA is confined inside narrower SWCNTs (diameter 3.12 nm and below). For a SWCNT of chirality indices (20,20) or diameter 2.71 nm, the average number of H-bonds is found to be ∼10 (0.7 H-bonds per base pair (bp)) up to approximately 80 ns and it decreases to ∼1 or 2 afterwards. With the increase in chirality indices or diameter of the SWCNT, the average number of H-bonds increases and reaches ∼36 (2.6 H-bonds per bp, similar to that of bulk dsDNA), for chirality indices (24,24) and above. For bulk dsDNA (dsDNA simulated without confinement), each CG base pair contributes three H-bonds, and each AT base pair contributes two H-bonds (see Figure 3). Hence the total number of canonical H-bonds for 14 central base pairs of the dsDNA used in this work is 36 (2.6 H-bonds per bp).

**FIGURE 4:**
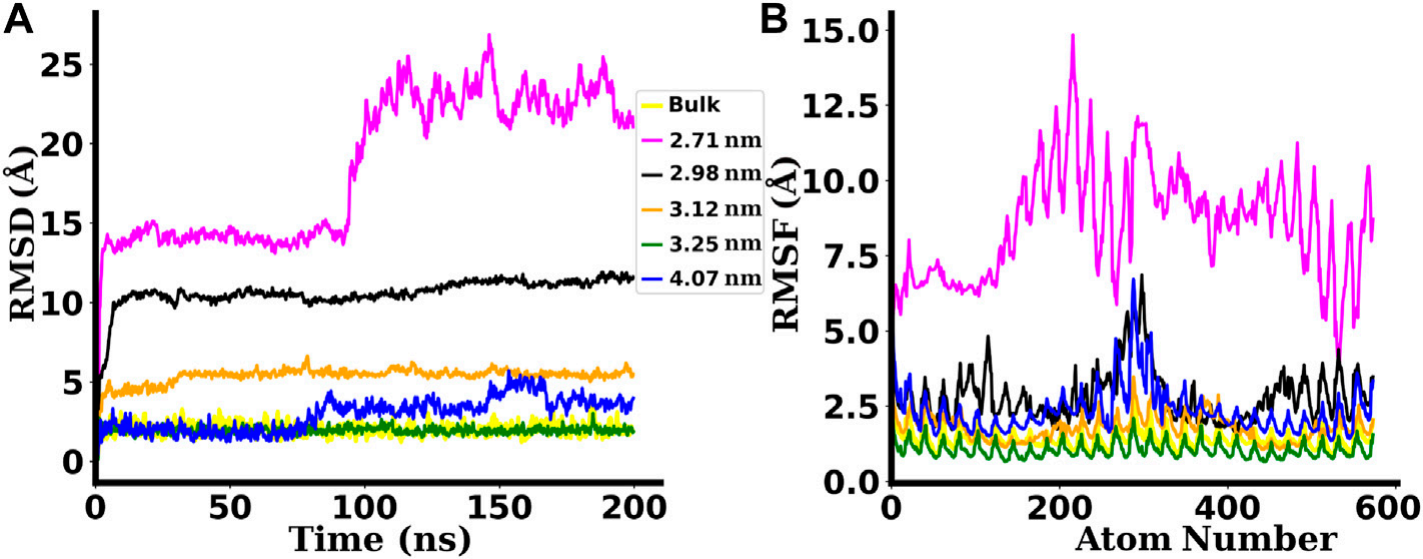
**(A)** RMSD and **(B)** RMSF plots for the dsDNA confined inside SWCNTs of different chirality indices. The labels used in the plots indicate the diameters of SWCNTs. Bulk is the case in which the dsDNA is simulated without any confinement. The backbone atoms of the dsDNA confined inside narrower SWCNTs with chirality indices below (24,24), i.e., 3.25 nm diameter are found to be denatured with higher RMSDs and RMSFs.

The non-native contacts between the dsDNA and the surface of SWCNT are found to be very high in the case of narrower SWCNTs. For chirality indices (24,24) corresponding to the diameter 3.25 nm and above, this value decreases sharply. For the widest SWCNT of diameter 4.07 nm with chirality indices (30,30), the number of non-native contacts rises after ∼80 ns of simulation time. A similar increase in the value of the RMSD is also seen in Figure 4A.

**FIGURE 5:**
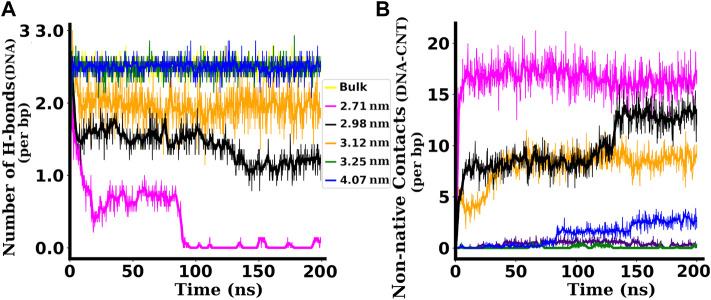
**(A)** Number of H-bonds per bp between inter-strand base pairs of dsDNA confined inside SWCNTs of different chirality indices. **(B)** Non-native contacts per bp between the dsDNA and SWCNTs of different chirality indices. For the dsDNA confined inside a SWCNT of chirality indices (23,23) corresponding to diameter 3.12 nm and below, the number of H-bonds is found to be highly reduced and the number of non-native contacts between the dsDNA and the SWCNT is highly increased. For the non-native contacts the distance cut-off of 3 Å is taken.

The significant decrease of H-bonds between the inter-strand bases of dsDNA (as shown in Figure 5A) and the abrupt increase of the number of non-native contacts between the dsDNA and the SWCNT surface in narrower SWCNTs (as shown in Figure 5B) signify that the dsDNA strands are denaturing and making contacts with the surface of the SWCNT. Such abrupt changes indicate melting of the dsDNA inside narrower SWCNTs. Interestingly, for the widest SWCNT of chirality indices (30,30) corresponding to the diameter of 4.07 nm, the increase of non-native contacts is due to the attraction and adsorption of the terminal base pairs of the dsDNA to the hydrophobic surface of the SWCNT as the dsDNA gets enough space to bend inside such wide SWCNTs.

The dehydration of confined dsDNA plays an important role in its denaturation: counter ions that are adequately hydrated stabilize dsDNA better than those that are not properly hydrated (Piškur and Rupprecht, 1995; Lau et al., 2005). To take this into consideration, we calculated the number of water molecules per bp within a cylindrical water shell of 3 Å thickness surrounding the dsDNA. Table 2 shows the hydration status of the dsDNA inside SWCNTs of different chirality indices. Inside a narrow SWCNT of diameter 2.71 nm (chirality indices (20,20)), the number of water molecules around the dsDNA is significantly low, with an average value of 37 ± 3 per bp. This situation changes when the diameter of the SWCNT is increased gradually. The average number of water molecules becomes 73 ± 2 per bp for diameter 3.25 nm (chirality indices (24,24)). For chirality indices (30,30), it becomes 87 ± 2 per bp, comparable to the value 89 ± 2 found in simulations of bulk dsDNA. Thus, inside narrower SWCNTs, dsDNA is not properly hydrated, which assists in its denaturation.

**TABLE 2:** Hydration status of dsDNA inside SWCNTs of different chiralites.

B1.45 chirality indices and diameter (*nm*)	No. of water molecules (per bp)	Chirality indices and diameter (*nm*)	No. of water molecules (per bp)
(20,20) 2.71	37 ± 3	(25,25) 3.39	77 ± 2
(22,22) 2.98	48 ± 3	(26,26) 3.52	80 ± 2
(23,23) 3.12	63 ± 2	(28,28) 3.79	84 ± 2
(24,24) 3.25	73 ± 2	(30,30) 4.07	87 ± 2
(No SWCNT) Bulk	89 ± 2		

Number of water molecules per bp within a cylindrical water shell of 3Å thickness surrounding the dsDNA.

### 3.3 Electrostatic Potential Maps and Axial Potential of Mean Force

To explain the dehydration of dsDNA inside narrower SWCNTs, we have computed the axial density of water inside the SWCNT as well as the free energy barrier experienced by water molecules at the both the ends of the SWCNT. Also, an isotropic distribution of counter ions around a dsDNA strand enhances its stability (Tan and Chen, 2006). To examine whether the confined dsDNA is surrounded by counter ions in an isotropic manner or not, electrostatic maps around the SWCNTs were computed. Our results for the electrostatic potential maps and the axial potential of mean force experienced by water molecules at entry points of the SWCNTs are discussed in this subsection.

**FIGURE 6:**
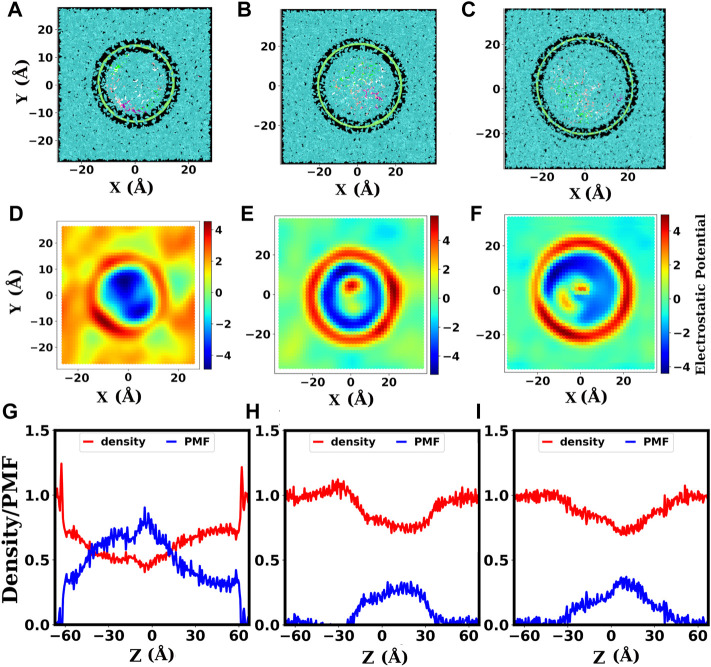
The electrostatic potential maps and axial density of water inside SWCNTs. **(A–C)**: Cross-sectional view (from the top) of the systems with dsDNA inside SWCNTs of diameters 2.71, 3.25, 4.07 nm, corresponding to chirality indices (20,20), (24,24), and, (30,30), respectively. The finite gap seen between the SWCNT and water surfaces depicts the hydrophobic nature of the SWCNT surface. **(D–F)**: Electrostatic potential maps for the systems with dsDNA inside SWCNTs of chirality indices (20,20), (24,24), and, (30,30), respectively. The color scale bar gives the electrostatic potential in units of *K*_*B*_ *T*/*e*. **(G–I)**: Axial density of water, showing the variation of the density of confined water along the length of the SWCNT, and the corresponding PMF for SWCNTs of chirality indices (20,20), (24,24), and (30,30), respectively. The PMF is in units of *k*_*B*_ *T*. The unit of axial density (*ϱ*) of water is g/cm^3^.

The electrostatic potential maps in Figures 6D–F show the distribution of counter ions inside and around the SWCNTs. Here, SWCNTs of chirality indices (20,20), (24,24), and (30,30) corresponding do diameters 2.71, 3.25, and 4.07 nm are taken as representatives for narrower, intermediate, and wider SWCNTs, respectively. Inside narrower SWCNTs (diameter less than 3.25 nm, Figure 6D), the electrostatic potentials are highly negative and distributed (because of the melted base-pairs) inside the SWCNT. Majority of the positive ions are not inside the pore and are distributed far away from the outer surface of the SWCNT. Inside the narrower SWCNTs, the denatured dsDNA is wrapped on the inner surface of the SWCNT (see Figure 8C). So, the negative potential is distributed non-uniformly over larger regions inside the SWCNT. For intermediate diameters of SWCNTs (from 3.25 nm to 3.79 nm, Figure 6E), the negative potential is more uniform inside the SWCNT because of stable dsDNA structure. Note that, in this case positive ions are isotropically distributed around the dsDNA near the wall of the SWCNT. Also, with the increase in diameter of SWCNT (diameter 3.25 nm or above), a significant number of positive ions enter into the SWCNT (see Figures 6E,F). In wider SWCNTs (diameter 3.79 nm or above), the end bases of dsDNA come in contact with the inner wall of SWCNT.

These electrostatic maps assist us in inferring that the dsDNA is adequately confined with uniform distribution of counter ions only beyond a threshold value of the diameter (3.25 nm) of the SWCNT. In the case of narrower SWCNTs (diameter 3.12 nm or below), the dsDNA gets denatured, and with denaturation, the DNA strands wrapped around the inner surface of the SWCNTs. Inside wider SWCNTs, the dsDNA gets enough space to bend, and the end residues come in contact with the hydrophobic surfaces of the SWCNT. Thus, the dsDNA is properly confined with an isotropic distribution of counter ions only for SWCNTs with intermediate diameters. Instantaneous snapshots presented in Figures 8C–E also substantiate these observations.

To explain the hydration status of dsDNA inside SWCNTs of different chirality indices, we computed the axial density *ϱ* of water inside the SWCNTs which describes the variation of density of water along the length of the SWCNT. The potential of mean force (PMF) for water is computed from the axial density distribution using the following formula (Trzesniak et al., 2007):

$$PMF=-k_{B}T\ln\varrho .$$ (7)

The free energy barrier experienced by water molecules to enter into the SWCNT can be obtained from the PMF. The unit of axial density (*ϱ*) of water is g/cm^3^. As *ϱ* is measured in g/cm^3^ and the bulk density of water is assumed to be 1 g/cm^3^, *ϱ* is equivalent to relative density.

The length of the SWCNTs used in our study is 120 Å  i.e., −60 Å–+60 Å  and there is a water buffer above and below its free ends. Figures 6G–I show the axial density and axial PMF profiles of water for SWCNT diameters 2.71, 3.25, and 4.07 nm corresponding to the chirality indices (20,20), (24,24), and (30,30), respectively. In these figures, we have taken the axial coordinate from −65 Å to +65 Å to take into account the external water buffer. As above, SWCNTs of chirality indices (20,20), (24,24), and (30,30) are taken as representatives for narrower, intermediate, and wider SWCNTs, respectively. Outside the SWCNTs, the density of water is ∼1 g/cm^3^ like in the bulk, as seen clearly for the water buffer. The water density inside the SWCNT suddenly decreases in the case of narrower SWCNTs (see Figure 6G) with diameter below 3.25 nm. This is caused by the narrower aperture and more hydrophobic nature of narrower SWCNTs.

With the increase in diameter, the entry of water molecules into the SWCNT becomes easier. The decrease in density at the middle of the SWCNTs occurs because the dsDNA occupies a larger fraction of space there. Therefore, the main points of interest are two ends of the SWCNT, i.e., the points with coordinate −60 and +60. At these points, the density of water is reduced in the case of the narrower SWCNTs (Figure 6G). No significant changes are found in SWCNTs with intermediate (Figure 6H) and wider diameters (Figure 6I).

We can see in Figure 6G that before the decrease near the ends of the SWCNT, the density shows a slight increment. This is because the water molecules form a cluster there due to the free energy barrier to entry. The free energy barrier for water molecules at the ends of the narrower SWCNT of 2.71 nm diameter is seen to be ∼ 0.5 in units of *k*_*B*_ *T*. For intermediate diameters and wider SWCNTs, there seem to be no significant barrier for the entry of water molecules. The significant barrier experienced by water molecules to enter into the narrower SWCNTs causes the dehydration of dsDNA which leads to its denaturation and melting.

### 3.4 Bonded and Non-bonded Interaction Energies

**FIGURE 7:**
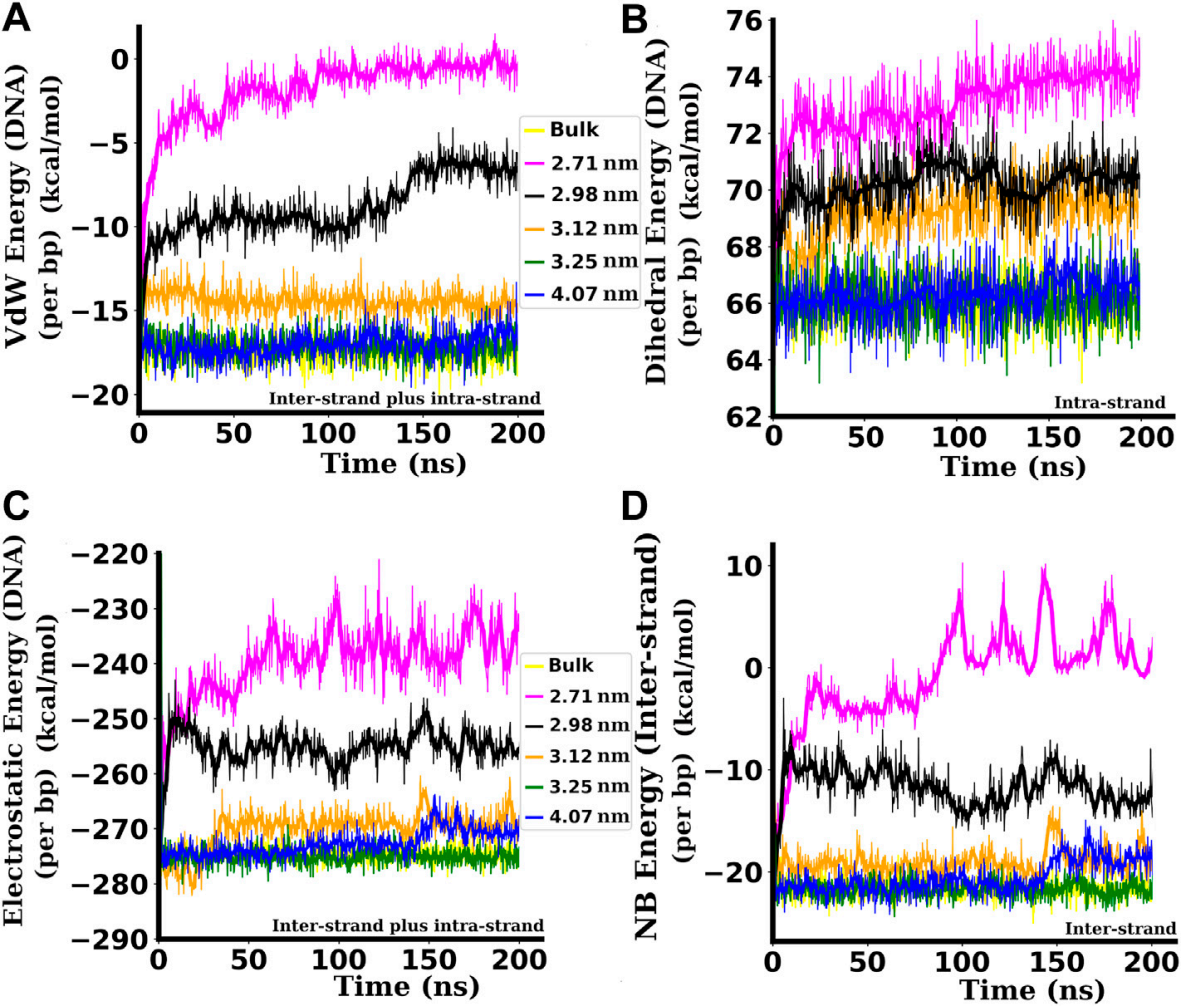
Different bonded and non-bonded interaction energies per bp between the bases of the dsDNA. **(A)** Inter and intra-strand Van der Waals (VdW) energies of the dsDNA. **(B)** intra-strand dihedral energies of the dsDNA. **(C)** Inter and intra-strand electrostatic energies of the dsDNA. **(D)** Inter-strand (without including intra-strand) non-bonded (NB) energies (VdW + Electrostatic) between two strands of the dsDNA. Attractive (−ve) interaction energies are decreased in magnitude, and repulsive (+ve) interaction energies are increased when the dsDNA is confined inside narrower SWCNTs. All the interaction energies presented here are in kcal/mol. Here, the inter-strand term is used for interaction energies between the residues of two different strands of dsDNA, and the intra-strand is used for the interaction energies between the residues of the same strand.

Weak, non-covalent interactions have an impact on dsDNA structure and stability. The primary non-bonded (NB) interactions relevant to dsDNA stability are Van der Waals (VdW) energy and electrostatic energy. Electrostatic interaction arises due to the asymmetric charge distribution in a molecule. Figure 7 shows different interaction energies per bp for the dsDNA confined inside SWCNTs of different chirality indices where the inter-strand term is used for interaction energies between the residues of two different strands of dsDNA and intra-strand is used for the interaction energies between the residues of the same strand. For VdW and electrostatic energies in Figures 7A,C, we have included both inter-strand and intra-strand interactions. Figure 7D includes only inter-strand interaction energies (VdW + electrostatic) without including intra-strand energies. The primary bonded interactions are bond stretching, angle bending, and dihedral interaction. The dihedral potential arises from repulsive interactions between overlapping bond orbitals and steric clashes between atoms in atomic planes and it is critical for the local structure changes of the biomolecule. For dihedral energies in Figure 7B, only intra-strand interaction is included since dihedral energy is the interaction energy between the atoms of atomic planes.

VdW interactions between base pairs on adjacent rungs of the double helix keep the bases at an optimum angle for tight packing of the double helix. When the dsDNA is confined inside a SWCNT of diameter 2.71 nm with chirality indices (20,20), the VdW energy per bp between the residues of the dsDNA is −1.86 ± 2.05 kcal/mol on the average. Inside a SWCNT of diameter 2.98 nm, corresponding to chirality indices (22,22), the VdW energy per bp is slightly higher in magnitude and is in the range of −8.77 ± 1.72 kcal/mol. Inside a SWCNT of diameter 3.12 nm, corresponding to chirality indices (23,23), the VdW energy per bp is −14.41 ± 0.63 kcal/mol on the average. For the diameter of 3.25 nm with chirality indices (24,24), the average VdW energy per bp is −17.23 ± 0.59 kcal/mol. For diameters of 3.25 nm and above, the VdW energy per bp settles to the average value of ∼ −17.25 kcal/mol, very similar to the value of bulk dsDNA (−17.40 ± 0.61 kcal/mol). The reduction of the attractive VdW energy (Figure 7A) pushes the dsDNA towards denaturation (see Figure 2) when it is confined inside SWCNTs of chirality indices below (24,24) or below diameter 3.25 nm.

**FIGURE 8:**
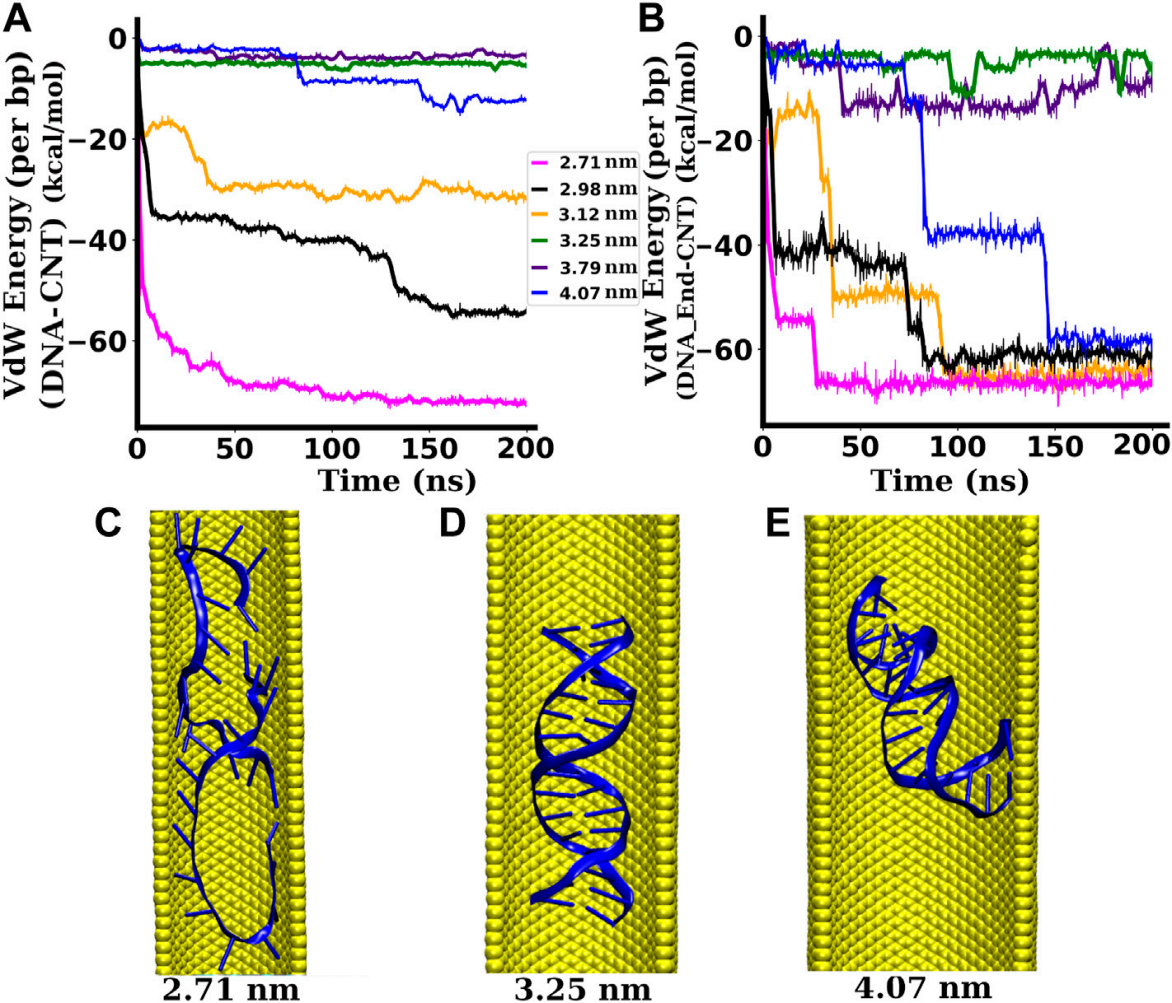
Interactions between the dsDNA and the SWCNT wall. **(A)** Interaction (VdW) energy per bp between the dsDNA and the SWCNT wall. **(B)** Interaction (VdW) energy per bp between the end base pairs of the dsDNA and the SWCNT wall. **(C)** Snapshot of the dsDNA inside a SWCNT of diameter 2.71 nm ((20,20) chirality indices). **(D)** Snapshot of the dsDNA inside a SWCNT of diameter 3.25 nm ((24,24) chirality indices). **(E)** Snapshot of the dsDNA inside a SWCNT of diameter 4.07 nm ((30,30) chirality indices). When the dsDNA gets space to bend, the hydrophobic end regions of the dsDNA get attracted towards the hydrophobic surface of the SWCNT while the central region remains nearly unaffected (Zhao and Johnson, 2007).

The electrostatic energies per bp are plotted in Figure 7C. The attractive or negative electrostatic energy between phosphates and other atoms of the dsDNA increases its stability. In this work, the attractive electrostatic energy is found to be smaller in magnitude for the dsDNA confined inside narrower SWCNTs of chirality indices below (24,24) corresponding to the diameter of 3.25 nm. Smaller negative values of energy means the structure is less stable and likely to be denatured. In wider SWCNTs, the attractive electrostatic energy increases in magnitude as the phosphate backbones are at adequate separation to minimize the repulsion between them, making the dsDNA more stable. When the dsDNA is confined inside a SWCNT of diameter 2.71 nm with chirality indices (20,20), the electrostatic energy per bp between the residues of the dsDNA is −238.85 ± 8.68 kcal/mol on the average. For the diameter of 3.12 nm with chirality indices (23,23), the average electrostatic energy per bp is −269.27 ± 9.11 kcal/mol. With diameters 3.25 nm and above, the electrostatic energy per bp settles to the average value of ∼−274 kcal/mol, almost the same (−274.26 ± 8.94 kcal/mol) as that of bulk dsDNA. The reduction in the attractive electrostatic energy (Figure 7C) also assists in the denaturation of the dsDNA confined inside narrower SWCNTs of diameter below 3.25 nm. In the case of a wider SWCNT of diameter 4.07 nm, we can see a slight reduction of the electrostatic energy between the bases of dsDNA after about 150 nanoseconds of simulation time. This is due to the adsorption of the end bases to the wall of the SWCNT, as shown in Figure 8E that leads to a slight unzipping of the end bases as described by Cruz et al. (Cruz et al., 2014; Cruz et al., 2015) and Nandy et al. (2012).

The intra-strand dihedral energies per bp of the dsDNA for different values of the confinement diameter (chirality indices of SWCNTs) are shown in Figure 7B. If the bases are frayed and come near any of the bases of the same strand, then the VdW distance decreases, and the phosphates of the two bases may overlap, causing repulsion. This increases the repulsive dihedral energy and decreases the VdW energy which drives the dsDNA towards instability. The instantaneous snapshots presented in Figure 2 show that the bases are frayed as described above, and hence the repulsion between the phosphates makes them structurally unstable and pushes the strands towards denaturation in a narrow confining environment. The repulsive or positive dihedral energy is found to be greater for the dsDNA confined inside narrower SWCNTs with diameters below the critical value of 3.25 nm, as shown in Figure 7B.

Along with the interaction energies of the whole dsDNA (taking both inter and intra-strand interactions into account), we have also computed the inter-strand non-bonded (NB) interaction energy (VdW and electrostatic) between the strands, i.e., without considering the interactions between the bases of the same strand. These non-bonded energies per bp between two strands, plotted in Figure 7D, are found to follow similar trends as those of the non-bonded energy plots for the whole dsDNA, with significantly lower non-bonded interaction energies between the strands for dsDNAs confined inside narrower SWCNTs of diameter less than 3.25 nm.

### 3.5 Pairwise Interaction Between dsDNA and SWCNT

The pair-wise interaction energies between the whole length of the dsDNA and the SWCNT surface as well as between the end bases of the dsDNA and the SWCNT surface are computed and plotted in Figures 8A,B, respectively. As the SWCNT is charge-neutral, the electrostatic energy between the SWCNT surface and the dsDNA is zero. Hence, in this case, the non-bonded interaction energy is the VdW energy only. For narrower SWCNTs of diameter less than 3.25 nm, the attractive interaction energy between the dsDNA and the SWCNT wall is significantly high in magnitude. With SWCNTs of diameter between 3.25 and 3.79 nm, the interaction energy is not significant and remains almost constant during the whole simulation. For a wider SWCNT of diameter 4.07 nm, the interaction energy is very small for the first few nanoseconds of simulation during which the distance between the dsDNA and the SWCNT remains large. After ∼ 80 ns, the magnitude of the interaction energy increases sharply (see Figure 8B) because the dsDNA is stretched and bent, and the end regions interact strongly with the hydrophobic surface of the SWCNT. With the availability of enough space to stretch and bend, there are additional VdW, hydrophobic, and hydrogen bonding interactions in the nanotube/water/DNA complex (Xue and Chen, 2005). The conformation of the dsDNA inside SWCNTs of narrower, intermediate, and wider diameters are depicted by snapshots in Figures 8C–E, respectively. With the availability of more space, the hydrophobic end bases of the dsDNA are attracted towards the hydrophobic SWCNT surface, while the hydrophilic backbone of the dsDNA is not strongly affected (Zhao and Johnson, 2007). As a result, the end regions of the dsDNA tend to bend towards the SWCNT surface, whereas the central region remains relatively unaffected. Such end-group attraction is not possible inside narrower SWCNTs because the dsDNA does not have sufficient space for significant bending. In the case of narrower SWCNTs (2.71–3.12 nm diameter), we observed a surprising increment of the attractive VdW energy after a few nanoseconds of simulation (see Figure 8A), reaching to −28.35 ± 4.59 kcal/mol per bp for 3.12 nm diameter and to −68.22 ± 6.53 kcal/mol per bp for 2.71 nm diameter. This is due to the fact that the dsDNA strands in these cases are melted due to the strong repulsive interaction between the negatively charged phosphate backbones and the strong hydrophobic interactions between the nucleobases and the wall of the SWCNT under strong confinement. In these cases, the dsDNA wraps on the hydrophobic surface of the SWCNT, as shown in Figure 8C. For the widest SWCNT of diameter 4.07 nm, the interaction energy between the end base pairs and the wall of the SWCNT is ∼ −7 kcal/mol per bp for initial a few nanoseconds and increases abruptly after that. The abrupt increment of the interaction energy between the dsDNA end bases and the SWCNT surface is due to the adsorption of the end bases of the dsDNA to the hydrophobic wall of the SWCNT. As the density of water inside narrower SWCNTs is very low, the water sheath between the dsDNA and the SWCNT surface becomes sparse. In such a region, the dielectric constant is lowered (Uematsu and Frank, 1980) and the interaction between the two hydrophobic regions, the wall of the SWCNT and the bases of the dsDNA, becomes stronger (Elder and Jayaraman, 2013). The sparse presence of water molecules between the dsDNA and the SWCNT surface reduces the number of competing H-bonds partners (Elder et al., 2015), which also helps to increase the interaction between the SWCNT and the dsDNA bases. This prompts the separation of dsDNA bases and ultimately results in the melting of the base pairs. In intermediate and wider SWCNTs, the water density increases around the dsDNA, due to which the number of competitive H-bonds is increased (Elder et al., 2015), which reduces the attraction between the bases of the dsDNA and the wall of the SWCNT. This prevents the base pairs of the dsDNA from melting.

All these observations indicate that the dsDNA’s melting or degree of denaturation is dependent on the diameter of the confining SWCNT. The threshold diameter of the SWCNT for proper confinement of the dsDNA without denaturation is found to be 3.25 nm, which corresponds to the chirality indices (24,24). This means that the dsDNA gets melted even at the physiological temperature of 300 K if the diameter of the confining SWCNT is less than 3.25 nm.

### 3.6 Effect of Chirality

**FIGURE 9:**
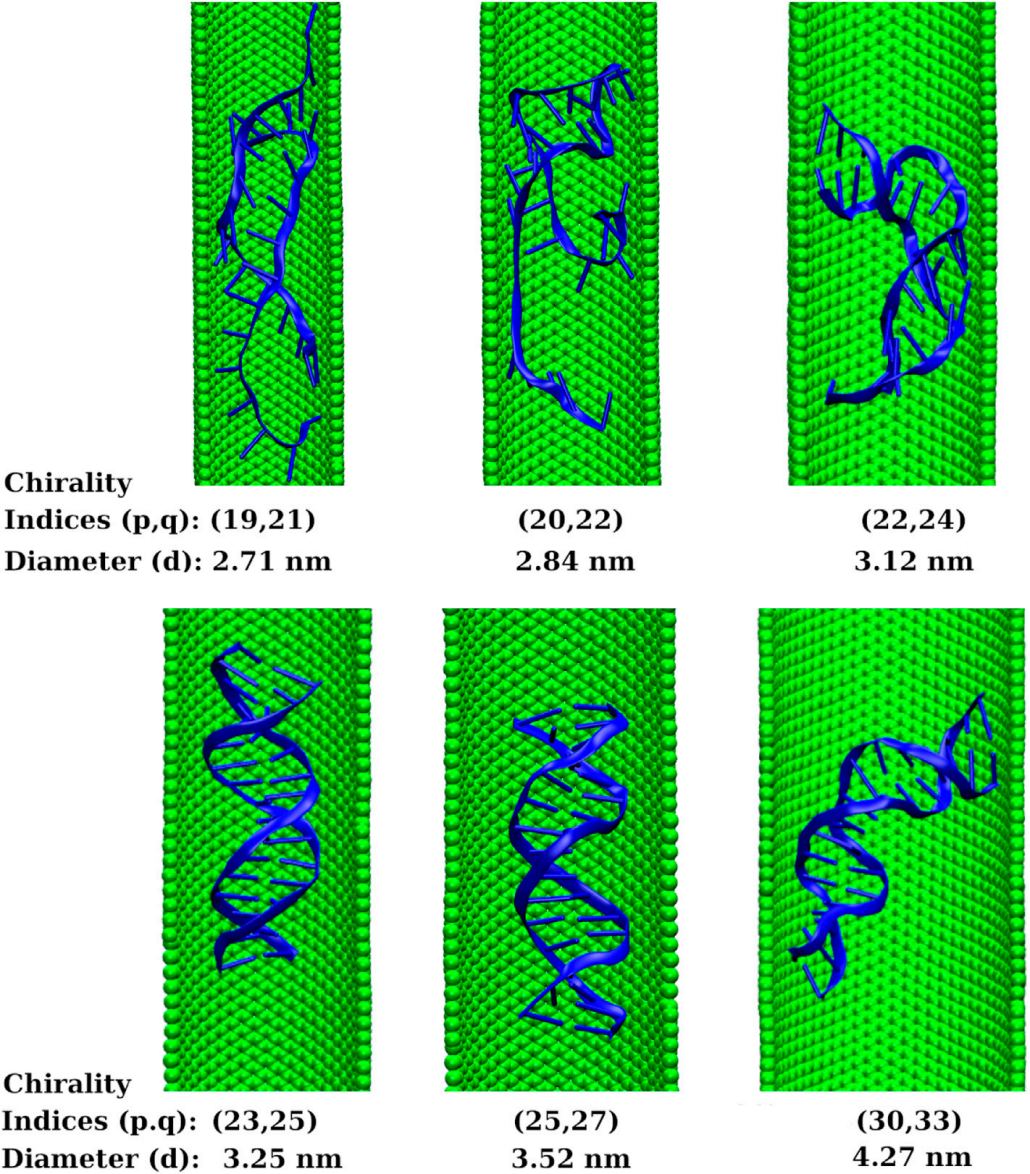
Instantaneous snapshots of dsDNA inside chiral SWCNTs of chirality indices (19,21), (20,22), (22,24), (23,25), (25,27), and (30,33) that correspond to diameters 2.71, 2.84, 3.12, 3.25, 3.52, and 4.27 nm, respectively. The snapshots depict that the threshold diameter of chiral SWCNT is also 3.25 nm for the proper confinement of the dsDNA inside the SWCNT.

The arm-chair (*p* = *q*) and zig-zag (*q* = 0) geometries are the achiral geometries (Dresselhaus et al., 2003). The chiral geometry is the one with *p* ≠ *q* (Dresselhaus et al., 2003). The arm-chair CNT shows metallic behaviour whereas the zig-zag and chiral CNTs show semiconducting behaviour (Yildirim Erbil, 2015). To examine whether the observed results are affected by the electronic properties of the confining environment, we did short simulations of 100 ns length with chiral SWCNTs of chirality indices (19,21), (20,22), (22,24), (23,25), (25,27), and (30,33) that correspond to diameters 2.71, 2.84, 3.12, 3.25, 3.52, and 4.27 nm, respectively. We found that the dsDNA confined inside the SWCNTs of chirality indices below (23,25) got melted. The dsDNA confined inside the SWCNTs of chirality indices (23,25) or above preserved its Watson-Crick geometry. The chirality indices (23,25) correspond to the diameter of 3.25 nm of the SWCNT, equivalent to the threshold diameter of arm-chair SWCNT that corresponds to the chirality indices (24,24). Inside wider SWCNT of chirality indices (30,33) corresponding to the diameter of 4.27 nm, the dsDNA is highly bent. It is due to the availability of enough space and the attraction of the hydrophobic end-bases of the dsDNA towards the inner wall of the SWCNT. These observations showed that the threshold diameter of chiral SWCNT is also 3.25 nm for the proper confinement of the dsDNA inside the SWCNT. The instantaneous snapshots for 100 ns are shown in Figure 9. It showed that chiral SWCNTs with *p* ≠ *q* also show similar results as that of arm-chair SWCNTs with *p* = *q*. It means the diameter dependence of the properties computed in this work is equally applicable to the SWCNTs other than arm-chair (metallic) SWCNTs also.

### 3.7 Mechanical Properties

**FIGURE 10:**
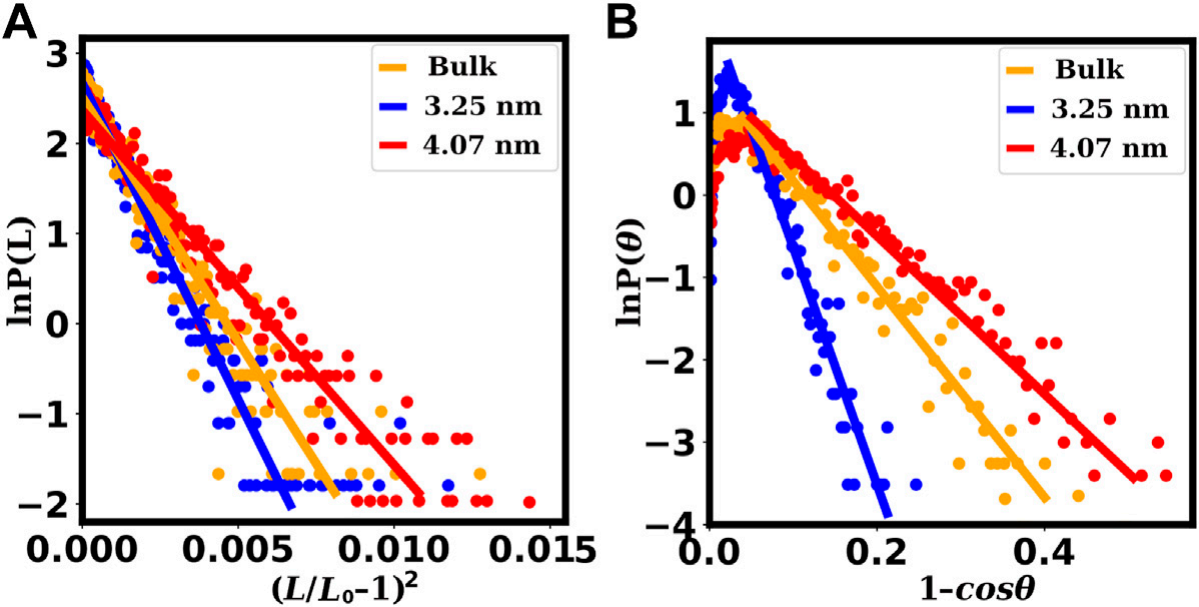
Fits for the computation of stretch moduli and persistence lengths of the dsDNAs confined inside SWCNTs of different chirality indices. The plots **(A)** for the stretch moduli are obtained from the fitting of Eq. 4, and the plots **(B)** for the persistence lengths are obtained from the fitting of Eq. 2. The red lines correspond to chirality indices (30,30) or 4.07 nm diameter, representing dsDNAs confined inside wider SWCNTs. The blue lines correspond to chirality indices (24,24) or 3.25 nm diameter, representing dsDNAs confined inside SWCNTs with intermediate diameters. The orange lines correspond to bulk dsDNA without confinement. In the case of dsDNAs confined inside wider SWCNTS, slopes of the lines are significantly smaller signifying surprisingly low values of the stretch modulus and the persistence length.

Various structural characteristics can be used to infer the dsDNA’s mechanical properties. The stretch modulus, persistence length, and torsional stiffness are three essential parameters that define the dsDNA’s elasticity. We computed these parameters for dsDNA confined within SWCNTs of large diameters for which the dsDNA is not denatured and adequately confined. Table 3 shows the values of these three elastic parameters of the dsDNA confined inside SWCNTs of diameter 3.25 nm (chirality indices (24,24)) and above. In Figure 10, we have presented linear fits to Eqs 2, 4 for three dsDNA duplexes (bulk, dsDNA confined inside a SWCNT of 3.25 nm diameter and dsDNA confined inside a SWCNT of 4.07 nm diameter) as representatives of free (bulk) dsDNA, dsDNA confined in SWCNTs of intermediate diameter, and dsDNA confined in wider SWCNTs. We did not compute the elastic characteristics of the dsDNA confined in SWCNTs with diameters below the critical value of 3.25 nm because it suffers from severe denaturation in such cases.

**TABLE 3:** Persistence length, stretch modulus, and torsional stiffness of the confined dsDNA.

Chirality Indices	Diameter in nm	Persistence Length (*l*_*p*_) in nm	Stretch modulus (*γ*_*G*_) in pN	Torsional stiffness(*C*) in pN.nm^2^
No SWCNT	Bulk	51.21 ± 2.05	1181.09 ± 48.21	473.84 ± 16.87
(24,24)	3.25	116.93 ± 4.58	1578.50 ± 55.85	762.02 ± 24.11
(25,25)	3.39	111.43 ± 4.93	1247.74 ± 43.64	549.30 ± 22.69
(26,26)	3.52	70.49 ± 2.27	1108.96 ± 50.73	535.95 ± 18.23
(28,28)	3.79	43.50 ± 2.44	966.38 ± 46.95	321.76 ± 14.41
(30,30)	4.07	32.49 ± 1.48	913.52 ± 43.86	313.43 ± 14.05
Experimental	Bulk	45 − 50	1000 − 1200	370 − 450

The last 50 ns trajectories are used to compute these mechanical properties. Persistence length (*l*_*P*_) is obtained from the slope of the straight line fitted to Eq. 2. The stretch modulus *γ*_*G*_ is obtained from the slope of the straight line fitted to Eq. 4, and the torsional stiffness (C) is obtained from the inverse of the variance of the twist angle *ϕ* using Eq. 5. Bulk is the case where the dsDNA is freely simulated without confinement.

In general, biopolymers such as DNA, RNA, protein, etc., can be characterized by their bending persistence length (*l*_*p*_), stretch modulus (*γ*_*G*_), and the torsional stiffness (*C*) (Noy et al., 2004). In our work, we found the persistence length for the bulk dsDNA to be 51.21 ± 2.05 nm, which is close to the experimental values of ∼45− 50 nm (Hagerman, 1997; Abels et al., 2005; Mathew-Fenn et al., 2008; Zoli, 2016; Liu et al., 2019). For the dsDNA confined inside a SWCNT of diameters 3.25 and 3.39 nm corresponding to the chirality indices (24,24) and (25,25), we found the persistence lengths to be increased to 116.93 ± 4.58 nm and 111.43 ± 4.93 nm, respectively. Structurally, the dsDNA inside SWCNTs of (24,24) and (25,25) chirality indices are highly rigid (see Figure 8D) because it does not have enough space to bend and tilt. In the case of a SWCNT of diameter 3.52 nm with chirality indices (26,26), the dsDNA has a bit more space for its dynamics, and hence the persistence length falls slightly to a value of 70.49 ± 2.27 nm.

As the diameter of SWCNT becomes larger, the persistence length of dsDNA decreases dramatically. For the dsDNA confined inside SWCNTs of diameters 3.79 and 4.07 nm with corresponding chirality indices (28,28) and (30,30), we found the persistence lengths to be 43.50 ± 2.44 and 32.49 ± 1.48 nm, respectively, smaller than that of bulk dsDNA. Structurally, the dsDNA inside SWCNTs of higher diameter, e.g. for chirality indices of (30,30), is found to be more flexible to stretch and bend (see Figure 8E) in comparison to dsDNAs in narrower SWCNTs and in water.

The torsional modulus of the bulk dsDNA is found to be 473.84 ± 16.87 pN nm^2^ in our study, which corresponds to a torsional persistence length of ∼114 nm that is quite close to the experimental values, ∼90− 110 nm, corresponding to a torsional modulus of ∼370− 450 pN nm^2^ (Herrero-Galán et al., 2013; Lipfert et al., 2014; Bao et al., 2017; Chhetri et al., 2022b). We found the torsional rigidity of dsDNA confined within SWCNTs of diameters 3.25 and 3.39 nm with corresponding chirality indices (24,24) and (25,25) to be increased to 762.02 ± 24.11 and 549.30 ± 22.69 pN nm^2^, respectively. This trend is similar to that found for the bending persistence length. For the dsDNA inside a SWCNT of diameter 3.52 nm with chirality indices (26,26), the torsional rigidity is slightly reduced to 535.95 ± 18.23 pN nm^2^, and it drops substantially as the diameter of the SWCNT is increased. The torsional rigidity of dsDNA confined inside SWCNTs of diameters 3.79 and 4.07 nm with corresponding chirality indices (28,28) and (30,30) is found to be 321.76 ± 14.41 and 313.43 ± 14.05 pN nm^2^, respectively.

A similar trend is seen in the values of the stretch modulus. For chirality indices (24,24) and (25,25) corresponding to diameters 3.25 and 3.39 nm, the stretch modulus of the dsDNA are found to be 1578.50 ± 55.85 pN and 1247.74 ± 43.64 pN, greater than the value, 1181.09 ± 48.21 pN for bulk dsDNA. The calculated value of the stretch modulus of bulk dsDNA is in good agreement with the available experimental values of ∼1000− 1200 pN for lower salt concentrations (Hagerman, 1997; Lipfert et al., 2014; Bao et al., 2017; Liu et al., 2019). But, with an increase in the diameter or chirality indices of SWCNT, the stretch modulus decreases gradually and becomes 1108.96 ± 50.73, 966.38 ± 46.95, and 913.52 ± 43.86 pN for chirality indices (26,26), (28,28), and (30,30) with corresponding diameters 3.52, 3.79, and 4.07 nm, respectively.

**FIGURE 11:**
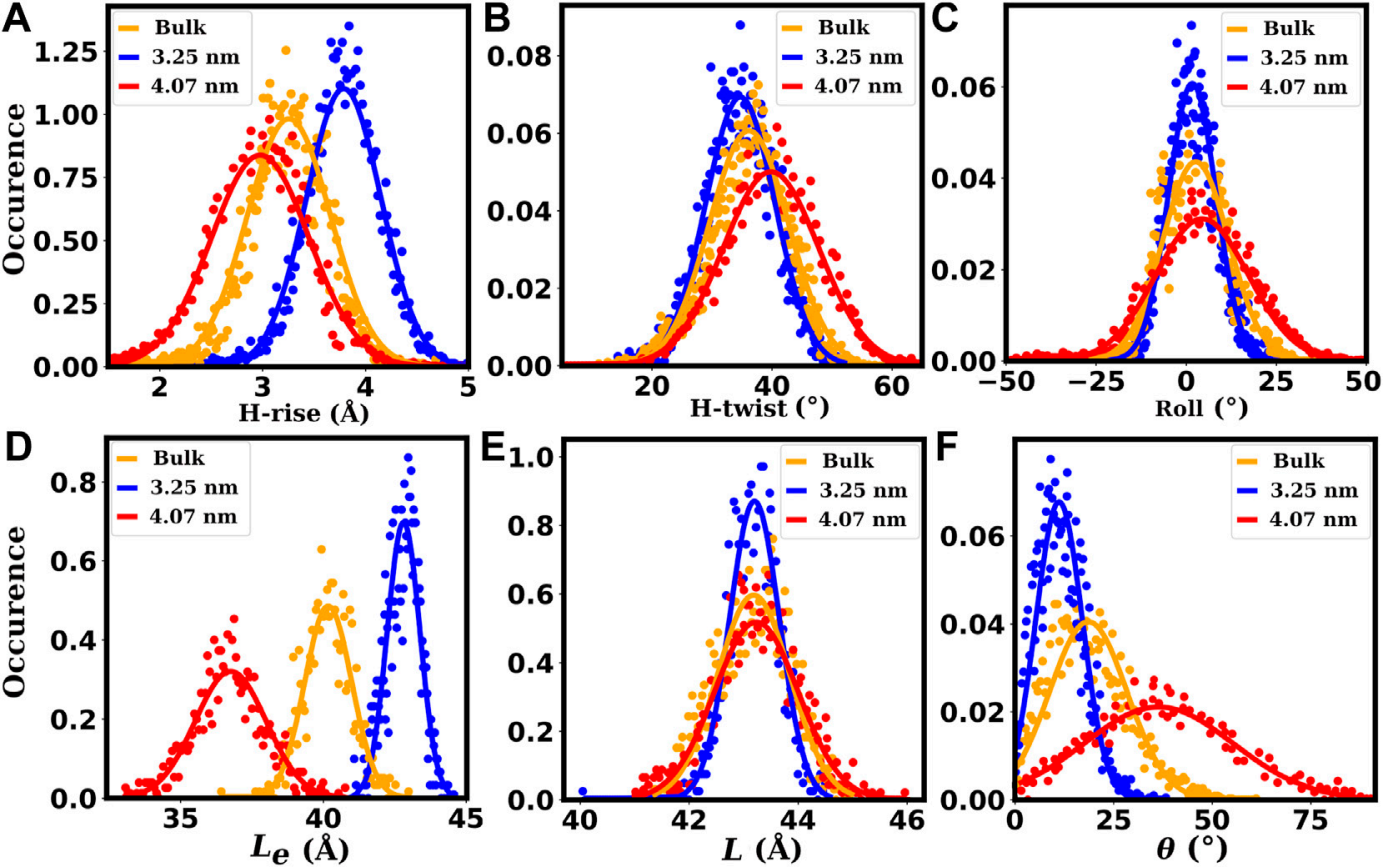
Some of the helical **(A–C)** and structural **(D–F)** properties of the dsDNAs. The plots **(A–C)** show the distributions of helical rise (H-rise), helical twist (H-twist) and roll parameters, respectively. The plots **(D–F)** show the distributions of the end-to-end distance (L_*e*_), contour length (L) and bending angle (*θ*), respectively. The red lines correspond to chirality indices (30,30) or 4.07 nm diameter of SWCNT, representing dsDNAs confined inside wider SWCNTs. The blue lines correspond to chirality indices (24,24) or 3.25 nm diameter of SWCNT, representing dsDNAs confined inside moderately wide SWCNTs. The orange lines correspond to bulk dsDNA without confinement.

To have more microscopic understanding of the mechanical properties presented in Table 3, we computed the distributions of some of the structural and helical parameters of the duplexes and plotted them in Figure 11. In Figure 11, we have presented the distribution plots for three dsDNA duplexes (bulk, dsDNA confined inside a SWCNT of 3.25 nm diameter and dsDNA confined inside a SWCNT of 4.07 nm diameter) as representatives of free dsDNA, dsDNA confined in moderately wide SWCNTs, and dsDNA confined in wider SWCNTs.

The roll parameter distribution of the base pairs and the bending angle (*θ*) distribution of the duplexes are shown in Figures 11C,F, respectively. Compared to bulk dsDNA, the bending is higher with higher fluctuation (standard deviation) in wider SWCNTs and smaller with low fluctuation in moderately wide SWCNTs. A similar trend is seen for the fluctuation of the roll parameter. The higher fluctuation of these parameters substantiates the higher bending flexibility (lower *l*_*p*_) of the dsDNA inside wider SWCNTs. Fluctuation of these parameters is lower inside moderately wide SWCNTs and indicates a higher bending rigidity of the dsDNA inside such narrower SWCNTs. The higher fluctuation of helical twist (H-twist) indicates a lower torsional stiffness of the dsDNA when confined inside wider SWCNTs (see Figure 11B). For the dsDNA confined inside SWCNTs of moderate diameter, the H-twist has lower fluctuation and implies a higher torsional rigidity. The helical rise (H-rise) parameter distribution of the base pairs, end-to-end distance (*L*_*e*_) distribution and contour length (L) distribution of the duplexes are shown in Figures 11A,D,E, respectively. Compared to the bulk dsDNA, the end-to-end distance is lower with higher fluctuation in wider SWCNTs and higher with low fluctuation in moderately wide SWCNTs. A similar trend is seen for the contour length and H-rise parameter fluctuations. The higher fluctuation of these parameters substantiates the higher stretching flexibility (lower *γ*_*G*_) of the dsDNA inside wider SWCNTs. Fluctuation of these parameters is lower inside moderately wide SWCNTs, implying a higher stretching rigidity of the dsDNA inside such narrower SWCNTs.

**FIGURE 12:**
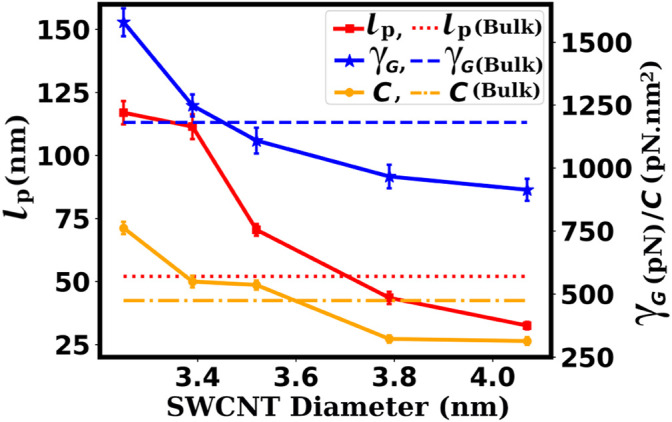
Variation of the persistence length (*l*_*p*_), stretch modulus (*γ*_*G*_), and torsional stiffness (*C*) of the confined dsDNA with respect to the diameter of the SWCNT. The dsDNA shows high rigidity for moderate confinement, and for weak confinement, the dsDNA shows high flexibility to bend, stretch, and twist. The dashed and dotted lines represent the values for the bulk dsDNA.

In Figure 12, we have plotted the variation of the persistence length, stretch modulus, and torsional stiffness of the confined dsDNA with respect to the diameter of the SWCNT. The high flexibility of dsDNA under weak confinement (wider SWCNT) is consistent with the results of Cifra et al. (Cifra et al., 2010). Under weak confinement, Cifra et al. found that dsDNA persistence lengths can reach about 60% of the bulk value. Also, they report the high rigidity of the strongly confined dsDNA. Inside moderately wide SWCNTs, higher rigidity of the duplexes to bend, twist, and stretch is expected due to the limited space available, which highly restricts the dynamics of the duplex. The decreases in the persistence length, torsional stiffness, and stretch modulus of the dsDNA inside substantially wider SWCNTs are, of course, related to the availability of more space, providing more flexibility to the dsDNA. But it is not clear why the values of these quantities are smaller than those for the bulk dsDNA. A full theoretical explanation for this behavior is missing currently. Below we try to provide some possible explanations.

The confinement and structure of the dsDNA inside SWCNTs of different diameters are depicted by snapshots in Figures 8C–E. With the availability of a wider space, the hydrophobic end groups of the dsDNA are attracted towards the hydrophobic SWCNT surface, while the hydrophilic backbone is not affected (Zhao and Johnson, 2007). As a result, the end regions of the dsDNA tend to bend towards the SWCNT surface, whereas the central region remains relatively unaltered. Such end-group attachment is not possible inside moderately wide SWCNTs since sufficient space is not available for significant bending. The dense buffer of mobile water molecules present between the dsDNA and SWCNT surfaces in wider SWCNTs may help to avoid permanent attachment of the dsDNA ends to the SWCNT surface (Lau et al., 2005). Thus, in the presence of mobile water molecules, the competition between the bending preferred by the hydrophobic end sections and the tendency of the central region to remain relatively unchanged makes the dsDNA more dynamic inside wider SWCNTs. The resulting increase in fluctuations is probably the main reason for the enhancement of the dsDNA’s bending, stretching, and torsional flexibility.

Mutual charge repulsion along the dsDNA backbones and attractive base stacking energies are thought to be major contributors to limiting the flexibility of the dsDNA (Williams and Maher III, 2000). In the strong bending regime, extensive softening of dsDNA-like biopolymers is known to occur (Drozdetski et al., 2019; Padinhateeri and Menon, 2013). This happens because the base stacking is weaker in highly bent dsDNA (Drozdetski et al., 2019; Williams and Maher III, 2000). Figure 7C shows a reduction of the attractive inter-strand electrostatic energy of the dsDNA inside a wider SWCNT of diameter 4.07 nm compared to the dsDNA inside a moderately wide SWCNT of diameter 3.25 nm, which suggests a weakening of the base stacking. The weakening of base stacking makes the orientational correlations between the dsDNA segments weaker (Cifra et al., 2010). It increases the inter-phosphate distances and hence reduces the repulsion between the backbones (Williams and Maher III, 2000; Cifra et al., 2010). Also, short dsDNA fragments exhibit surprisingly high flexibility (Yuan et al., 2008; Zoli, 2018), and such high flexibility comes mostly from the shearing of the bases of the molecule (Fathizadeh et al., 2012). Thus, weaker correlations between the internal segments of highly bent dsDNA in weak confinement (wider SWCNT) can be another reason of the enhancement of the flexibility of the dsDNA.

## 4 Conclusion

The cell membrane acts as an impenetrable barrier for molecules like DNA and RNA that contain negatively charged phosphate backbones. Several ways have been devised to allow nucleic acids to pass through the cell membrane, including the packaging of nucleic acids inside a physical structure that creates temporary holes in the membrane, allowing nucleic acid molecules to enter the host cells directly. SWCNTs are considered to be promising options for doing this. Numerous experimental, theoretical, and simulation works have shown that the biomolecules can be confined inside SWCNTs with 2.7 nm radius and MWCNTs with 3–4 nm radius at relatively high temperatures of 350–400 K. In this work, we simulated dsDNA molecules that had been inserted into SWCNTs of different diameters and studied their physical and energetic features in the constrained geometry at the physiologically relevant temperature of 300 K.

We found that a dsDNA can be confined adequately without being denatured only after a threshold value of the diameter of the SWCNT. Below this threshold diameter, the dsDNA is found to be denatured and melted even at the physiologically relevant temperature of 300 K. Our simulations with chirality indices (24,24) to (30,30) of SWCNTs at 300 K found the critical diameter to be 3.25 nm, corresponds to chirality indices (24,24). The H-bonds count showed that significant number of H-bonds between the dsDNA bases are broken below this critical diameter. H-bonds provide stability to dsDNA to keep the strands of dsDNA intact. Large reduction in the number of the H-bonds of dsDNA inside narrower SWCNTs make the strands to be separated, resulting in the melting of the base pairs.

Analyses of VdW and other inter-base interactions also showed a severe reduction of the magnitudes of the attractive VdW and electrostatic energies for dsDNAs confined in SWCNTs with chirality indices below the (24,24) that corresponds to diameter 3.25 nm. The dihedral energy computation showed that dsDNA bases are frayed, resulting in repulsion between the planes and an increase in the repulsive dihedral energy that drive the dsDNA base pairs towards melting. The pair-wise VdW interaction energy between the dsDNA and the SWCNT increased significantly in magnitude below the critical diameter 3.25 nm of the SWCNT. This is a consequence of dsDNA melting inside SWCNTs with diameters below the critical value. With the denaturation of the dsDNA, the strands of dsDNA wrapped on the hydrophobic surface of the SWCNT, thereby generating a sharp rise in the magnitude of the VdW energy.

Through electrostatic map, we saw an isotropic distribution of counter ions around the dsDNA when it is confined inside SWCNTs of relatively wide diameters. This assists in stabilizing the dsDNA strands, as counter ions under appropriate hydration play an important role in stabilizing dsDNAs. For narrower SWCNTs, the counter ions are found to move away from the SWCNT surface. This helps the denaturing of the dsDNA. In addition to this, the dsDNA is not fully hydrated in narrower confinement. The sparseness of water molecules between the dsDNA and the SWCNT surface reduces the number of competing H-bonds partners, which helps to increase the interaction between dsDNA bases and the SWCNT wall. This results in the loss of H-bonds between the bases of dsDNA. So, inadequate hydration prompts the denaturation of dsDNA inside those narrower SWCNTs.

Computation of the mechanical properties of dsDNA that is not denatured when confined inside SWCNTs of chirality indices (24,24) and above showed that the persistence length and the torsional stiffness of dsDNA increase sharply for the chirality indices (24,24) to (26,26), corresponding to diameters 3.25 and 3.52 nm, respectively. The persistence length becomes about twice that of a free dsDNA in this range, and the torsional stiffness becomes larger by a factor of about 1.5. When analyzed for chirality indices (28,28) and (30,30) or diameters 3.79 and 4.07 nm, we found that the persistence length and the torsional stiffness fall dramatically such that their values are even smaller than those of bulk dsDNA. The stretch modulus also follows a similar trend: first increasing with an increase in chirality indices and then decreasing sharply after diameter 3.79 nm (chirality indices (28,28)) and above. Such initial increase and then unexpected decrease are interesting and can be explained on the basis of the work of Zhao and Johnson (Zhao and Johnson, 2007). According to Zhao and Johnson (Zhao and Johnson, 2007), with the availability of a wider space, the hydrophobic end groups of the dsDNA are attracted towards the hydrophobic SWCNT surface, while the hydrophilic backbone is not affected. As a result, the end regions of the dsDNA tend to bend towards the SWCNT surface, whereas the central region remains relatively unaltered. Our analysis of pairwise interaction energies between the dsDNA and the SWCNT surface also shows similar results. So, we assume that, inside wider SWCNTs containing a large number of water molecules, the balance between the end regions that favor bending and the central region that tends to remain unaltered makes the dsDNA more dynamic, thereby enhancing the bending, twisting, and stretching flexibility of the dsDNA. Also, the weakening of base stacking which results in weaker correlations between the internal segments of highly bent dsDNA inside wider SWCNT, is expected to enhance the flexibility of dsDNA.

Thus, we found the denaturation and melting of dsDNA inside SWCNTs to be diameter dependent, with a critical diameter of 3.25 nm corresponding to chirality indices (24,24) of the SWCNT. Below this critical diameter of SWCNT, the dsDNA is denatured, and above it, the dsDNA retains its native structure. The elastic properties of the dsDNA in cylindrical confinement inside SWCNTs are also found to be diameter dependent. Inside moderately wide SWCNTs, the confined dsDNA is highly rigid to bend, stretch and twist, whereas inside wider SWCNTs, the elastic properties change surprisingly, making the dsDNA highly flexible.

Using cylindrical single-walled CNTs, we found the melting of dsDNA in strongly confined environments. To assess the generality of the current findings, different shapes of confining nano-surfaces of different materials, such as graphene, carbon, boron nitride, silicon and MoS_2_, both single-walled and multi-walled, can be the subjects of future research to conduct similar investigations.

## Data Availability

The original contributions presented in the study are included in the article/Supplementary Material, further inquiries can be directed to the corresponding author.
